# The children’s emotional speech recognition by adults: Cross-cultural study on Russian and Tamil language

**DOI:** 10.1371/journal.pone.0272837

**Published:** 2023-02-15

**Authors:** Elena Lyakso, Nersisson Ruban, Olga Frolova, Mary A. Mekala

**Affiliations:** 1 The Child Speech Research Group, St. Petersburg State University, St. Petersburg, Russia; 2 School of Electrical Engineering, Vellore Institute of Technology, Vellore, India; 3 School of Information Technology and Engineering, Vellore Institute of Technology, Vellore, India; University of California Los Angeles, UNITED STATES

## Abstract

The current study investigated the features of cross-cultural recognition of four basic emotions “joy–neutral (calm state)–sad–anger” in the spontaneous and acting speech of Indian and Russian children aged 8–12 years across Russian and Tamil languages. The research tasks were to examine the ability of Russian and Indian experts to recognize the state of Russian and Indian children by their speech, determine the acoustic features of correctly recognized speech samples, and specify the influence of the expert’s language on the cross-cultural recognition of the emotional states of children. The study includes a perceptual auditory study by listeners and instrumental spectrographic analysis of child speech. Different accuracy and agreement between Russian and Indian experts were shown in recognizing the emotional states of Indian and Russian children by their speech, with more accurate recognition of the emotional state of children in their native language, in acting speech vs spontaneous speech. Both groups of experts recognize the state of anger via acting speech with the high agreement. The difference between the groups of experts was in the definition of joy, sadness, and neutral states depending on the test material with a different agreement. Speech signals with emphasized differences in acoustic patterns were more accurately classified by experts as belonging to emotions of different activation. The data showed that, despite the universality of basic emotions, on the one hand, the cultural environment affects their expression and perception, on the other hand, there are universal non-linguistic acoustic features of the voice that allow us to identify emotions via speech.

## Introduction

There are innate (basic) and acquired emotions during life activities. Innate emotions have a similar pattern of manifestation regardless of socio-cultural rules, but their recognition depends on many factors [1]. One of the main difficulties in describing emotions lies in the fact that emotion manifests itself simultaneously in internal experiences, and in external behavior, reflected in postures, mimic expression, voice characteristics, and emotive words. There is an evidence of universal facial expressions for basic emotions such as happiness, sadness, anger, fear, surprise, and disgust [1–4] and vocal expressions for basic emotions [5, 6]. Decades of research have shown that the voice is a powerful tool for expressing emotions. The study of recognition of human emotions by voice has a long history [7] and at present, it is widely used in the creation of automatic systems that can recognize and model human emotions [8–10], and in a broader aspect of determining the information contained in emotional speech prosody [11].

Recognition of emotional states depends on many factors, such as socio-cultural rules [12], age of the speaker and listener [13], gender [14], types of emotion. Improvement was shown in vocal emotion recognition from adolescence to adulthood (19–35 years) with smaller improvement in accuracy between childhood (8-to-10 year old) and adolescence (11- to-13 year old). Accuracy improved with age for all emotions (anger, happiness, sadness, fear, and neutrality) in the native language (English), but in the non-native language (Spanish, Chinese, and Arabic) the improvement was not observed for certain emotions (e.g., anger) [15]. A recent study showed that the accuracy of recognizing vocal emotions improved from childhood to early adulthood, and decreased in the elderly [16].

The emotions in voice play a significant role in communication without visual contact when fast recognition of the interlocutor’s emotions is important. Anger, sadness, fear, and neutral expressions are recognized more accurately at short gate intervals than happiness, and particularly disgust. However, as speech unfolds, recognition of happiness improves significantly by the end of the utterance, and fear is recognized more accurately than other emotions [17]. The most difficult task when studying the emotions reflected in the voice is in cross-linguistic research. A meta-analysis of 37 cross-cultural studies of emotion recognition on speech prosody and non-linguistic vocalizations, including emotion expressers from 26 cultural groups and emotion receivers from 44 different cultures, found that a wide range of positive and negative emotions are recognized with high accuracy in intercultural conditions. The second main conclusion was the determination of the influence on the recognition of the diversity of cultures expressing and perceiving [6].

Research into the recognition of non-verbal emotional vocalizations, such as screaming and laughing, in two very different cultural groups—Western participants (English) and people from culturally isolated villages in Namibia, showed that vocalizations that convey basic emotions (anger, disgust, fear, joy, sadness, and surprise), were recognized bidirectionally [5]. These data support hypothesis that vocal emotional expressions of the main affective categories are manifested similarly in very different cultures. Shuar individuals from Amazonian Ecuador were able to reliably identify happy, angry, fearful, and sad vocalizations produced by American native English speakers by matching emotional spoken utterances to emotional expressions portrayed in pictured faces [18]. The aim of another study [19] was to test the hypothesis that norms in interdependent cultures around socially disengaging emotions may influence nonverbal vocal communication of emotions. To test this hypothesis, a cross-cultural experiment was performed in which Dutch and Japanese listeners categorized and rated Dutch and Japanese vocalizations expressing nine emotions including anger and triumph, two socially disengaging emotions. The results of this study demonstrate that Japanese vocalizations of socially disengaging emotions, especially anger, are challenging to interpret for Western listeners. Consistent with previous studies both Dutch and Japanese listeners were generally able to recognize emotions expressed by both in-group and out-group members at the above chance level was showed [20]. Cross-cultural studies often indicate that while basic emotion recognition is universal and more accurate when speakers and receivers come from the same culture compared to other crops [4, 5, 21]. Other researchers [22] tested the hypothesis about the effect of prior cognitive experiences on judgment when listening to vocal material. Professional actors from Australia and India vocally portrayed different emotions (anger, fear, happiness, pride, relief, sadness, serenity, and shame) by enacting emotion eliciting situations. When listening to vocal material, Australian and Indian listeners had to try to imagine what event the speaker was reacting to in the recording and then answer six questions about an imaginary event (presented simultaneously on-screen) associated with each recording. Overall, the results indicated that appraisal ratings were relatively consistent across cultures. The authors of the paper [22] conclude that few group differences emerged, which suggests that the perceived appraisal profiles are largely universal.

The frame of Dialect theory argues that although emotional communication is culturally universal, it is characterized by accents that reflect the distinct cultural style for expressing nonverbal cues [23]. Cross-cultural studies on the recognition of emotions by voice and speech of Indian and European participants are unitary. Cross-cultural studies of emotional prosody recognition by Hindi and Canadian English listeners showed that in each language state, native listeners were faster and more accurate in recognizing emotions than non-native listeners [12]. In this study, the participants were Indians who were born and raised in different parts of India and all spoke Hindi at home with both parents as children; each moved as a youth to Montreal, Canada to study or work, i.e. they all spoke English and Hindi. They were listening to pseudo-statements conveying the four basic emotions, expressed in English and Hindi.

As far as is known, there are no cross-cultural data on the recognition of the basic emotions via speech by native Russian and native Tamil listeners. Russian is one of the East Slavic languages which belong to the Indo-European family. Tamil is one of the classical languages in the world [24]. Tamil is a language of the Tamil-Kannada group of Eurasian languages which belongs to the Dravidian family. Both languages have an ancient origin, and the countries where these languages are used have a centuries-old original culture. Cross-cultural recognition of emotions from the voice and speech of children speaking Russian and Tamil is an even more difficult challenge in comparison with the cross-cultural recognition of emotions via speech of adults. The results of recognition of basic emotions in the voice of children by native speakers of two different language groups will be able to supplement existing knowledge about cross-cultural perception of emotions. The younger school age of children is the most interesting for research, since, on the one hand, children have not yet fully mastered the cultural traditions of emotional manifestations, and on the other hand, their development in the appropriate cultural environment has already influenced their emotional sphere.

The hypothesis of the study is to test the assumption that basic emotions in the speech of Russian and Indian children will be recognized by native speakers of Russian and Tamil languages, but dialect-specific recognition features will be revealed. The goal of the study was to compare the cross-cultural recognition of four emotions “joy–neutral (calm state)–sad–anger” in the spontaneous and acting speech of children aged 8–12 years across Russian and Tamil languages. The research tasks were to examine the ability of Russian and Indian experts to recognize the state of Russian and Indian children by their speech, determine the acoustic features of correctly recognized speech samples, and specify the influence of the expert’s language on the cross-cultural recognition of the emotional states of children.

## Method

### Listeners

Participants were listeners speaking the Russian and Tamil languages, who were invited to listen to speech samples of Russian and Indian children, pronounced in different emotional states. A study with two groups of participants was conducted to find out if the native language of the listener influences the recognition of the emotional state of children reflected in their speech. The results of the assessment of the listener’s groups were subjected to cross-comparisons in subsequent analyses.

The native language of the Indian listeners is Tamil, and the native language of the Russian listeners is Russian. None of the Russian participants knew Tamil, and the Indian listeners did not know Russian. The common language for the listeners of the two countries was English. Both listeners groups did not visit their counterparts’ countries. Their knowledge of the culture of the camp of researchers of another language group is based on literary sources and feature films and documentaries. In total, 26 listeners participated in the experiment. Both native Russian (n = 13, age Mean ± SD 35.8 ± 12.6 years) and native Tamil (Indian) (n = 13 age, 37.6 ± 10.7 years) listeners have special education in speech sciences and professional experience in the field of speech science–experts (12.8 ± 8.8 y–for Russian listeners; 13.7 ± 9.2 y–for Indian listeners).

### Speech data collection

Emotional speech recordings were collected from 30 children aged 8–12 years: 12 Russian-speaking children (born and living in St. Petersburg, Russia), 18 Tamil-speaking children (born and living in Vellore, India). The Tamil language is mainly slang influenced [25]. In this study, we have used Chennai / Vellore based common slang of the Tamil language speaking children.

The place of recording of child’s speech and behavior was the laboratory. All procedures were approved by the Health and Human Research Ethics Committee of Saint Petersburg State University (Russia) and written informed consent was obtained from parents of the child participant. Signed consent forms are filled by the parent of each Indian child. The entire procedure was supervised and supported by a Senior Pediatrician, who is part of the research team.

The study was carried out according to the common protocol. Two types of speech were used: spontaneous speech and acting speech.

Spontaneous speech—The dialogue between the child and the experimenter was used to obtain the child’s spontaneous speech. We assumed that the semantically different questions could provoke different emotional states in the children [26]. The standard set of experimenter’s questions addressed to the child was used. The experimenter began the dialogue with the request to say your name and age. Then the experimenter consistently asked questions:

- Do you like to go to school? What do you like in school (classes or playing with friends)? What are your favorite tasks? Why? Do you have any hobbies? What are your favorite movies, cartoons, books, games (computer/desktop/mobile)?
- What do you dislike at the school? What subject do you dislike the most? Why? Are you angry with anyone? How often do you get angry? If there is a quarrel, do you fight right away or do you first find out the cause of the conflict?
- Do you know what sadness is? How do you feel if you are sad? When (in what situations) do you think a person experiences sadness?

Acting speech–Before recording the speech material, children were trained in pronouncing words, words and phrases, and meaningless texts demonstrating different states–neutral (calm)–joy–sadness–anger. The set of words and set of words and phrases (Emotional words and emotional words & phrases) both for Russian and Indian children: The speech materials reflecting different emotional states “joy–neutral–sadness–anger” were selected according to the lexical meaning of words /joy, beautiful, cool, super, nothing, normal, sad, hard, scream, break, crush/ and phases /I love when everything is beautiful, Sad time, I love to beat and break everything/. The children should pronounce the speech material, manifesting the emotional state. This task was designed to show how children could demonstrate different states in vocal expressions. One of the approaches to the study of emotional speech is the use of meaningless sentences. Meaningless sentences are pseudo sentences (semantically meaningless sentences that resembled real sentences) as other researchers use [27], a set of semantically neutral sentences and derived pseudo sentences [28]. Russian children had to demonstrate acting skills when pronouncing a meaningless text–a fragment of “Jabberwocky”, the poem by Lewis Carroll [29], the meaningless text (sentence) by L.V. Shcherba “glokaya-kuzdra” (1930) [30]; Indian children spoke the meaningless text about Grandpa [31] and Tamil meaningless phrases.

For each child, the total recording time was 30–40 minutes. The recordings of speech of children were made by the “Marantz PMD660” recorder with external microphone “SENNHEIZER e835S” with the following settings: the sampling rate was set to 16,000 Hz and the mono audio channel was used in all the recording sessions. Parallel with the recording of the speech, the child’s behavior and mimic expression were recorded using a video camera “SONY HDR-CX560E”. Video recording is carried out in studies of the emotions’ manifestation in speech and facial expressions [32, 33] and, along with the recording protocol, is used by experts when annotating speech material that reflects different emotions. The recording was carried out in rooms without special soundproofing. The distance from the child’s face to the microphone did not exceed 50 cm (30–50 cm). Speech and video records for children are included in the child speech corpus. All speech files were stored in.wav format, 44100 Hz, 16 bits per sample.

### Data analysis

The annotation of the child’s emotional speech material was made by four categories (based on video records and the recording situation protocol) “joy–neutral–sadness–anger” by two Russian speaking speech specialists for Russian children and by two Tamil speaking specialists for Tamil speaking children. These specialists did not participate in subsequent perceptual experiments. The speech sample was considered as attributed to the corresponding emotion only when two experts gave the same answers.

The study includes two methods: perceptual study and instrumental spectrographic analysis of child speech. Two experimental perceptual studies were carried out. The stimulus material contained words and phrases of children arranged in test sequences (Table 1). The different stimulus material was selected to determine the impact of a type of speech material on emotions recognition. An unique voice number is given for each speech sample (in English).

**Table 1:** The stimulus material.

	Type of speech	Language	Test, n	Speech samples, n
**Study 1**	Spontaneous: Words & phrases	Russian	2	90
		Tamil	2	90
**Study 2.1**	Acting: Emotional words	Russian	1	44
		Tamil	1	44
**2.2**	Acting: Emotional words & phrases	Russian	1	16
		Tamil	1	16
**2.3**	Acting: Meaningless texts	Russian	1	16
		Tamil	1	16

Each test was listened by 10 experts. For all the studies, the experts indicated in the questionnaire the information about themselves: gender, age, and experience in interacting with children. The task for experts was to identify four classes: “joy—neutral—sadness—anger”. There was no preliminary training for the experts. The experts listened to each test once. Experts listened to the test sequences through headphones “SENNHEIZER”. The speech intensity level in the tests during playback was 60–70 dB. The experiment was carried out in a laboratory, the noise level in which did not exceed 20 dB.

Spectrographic analysis of children’s speech was carried out in the Cool Edit Pro sound editor and Praat v. 6.1.42 [34]. The temporal and spectral characteristics of speech were automatically calculated, based on the algorithms implemented in the Cool Edit Pro sound editor, the intensity (energy) was automatically calculated in Praat. For all speech samples included in the test sequences, the duration (ms) of a word, phrase or utterance was determined; by word, phrase, utterance: pitch values (F0)—average, F0max, F0min (Hz), and intensity values E0 (dB). F0 is the main characteristic of the voice, resulting from the swaying of the vocal folds. F0 statistics are one of the most important features that correlate with emotional vocal expressions. A higher and wider range of F0 [13, 32, 35, 36] and energy [35] are usually associated with high-arousal emotions compared to neutral speech. For each utterance, the range of F0 was calculated by subtracting the minimum F0 from the maximum F0 values: F0 range = F0[max-min], the ratio of intensities corresponding to F0max—E0max and F0min = E0min (dB) normalized with respect to E0 average—E0max / E0, E0min / E0; the ratio E0max / E0min was calculated.

Statistical data analysis was carried out in the “Statistica 10” using non-parametric tests: Mann-Whitney test, Spearman correlation (p < 0.05), Regression analysis, and Multiple Regression analysis. The Spearman correlations were validated by Regression and Multiple Regression analysis. In the perceptual experiment, Mann-Whitney test was used for comparing the accuracy of two groups of experts (Russian and Indian) classifying the emotional state of children. Confusion matrices for perceptual experiments were prepared. A confusion matrix (error matrix) is used to describe the performance of a classification model. It is a table, the rows of which correspond to the given (projected) classes, the columns correspond to the actual values (real classes). We counted recall, precision, F-1 score for each emotion, Unweighted Average Recall (UAR)—for all emotions [37]. The precision within the class is the proportion of samples that actually belong to this class, relative to all samples that were assigned to this class. The recall is the proportion of samples found by the classifier belonging to a class relative to all samples of this class in the test sample. The F1-score means the harmonic mean between precision and recall. Agreement between experts of the same language group and different groups is assessed using the Cohen kappa statistic (k) [38, 39]. Relative strength of agreement was associated with kappa statistics: slight (0.00–0.20), fair (0.21–0.40), moderate (0.41–0.60), substantial (0.61–0.80), and almost perfect (0.81–1.00) [40]. In stimulus material description, Man-Whitney test was used for comparing acoustic features of speech samples between Russian and Indian children and revealing the influence of child’s gender on acoustic features. The aim of Regression analysis was to explore the correlation between a child’s emotional state, language and acoustic features of speech. The aim of Multiple Regression analysis was to reveal the correlation between types of emotional acting speech of children (words, words and phrases, meaningless texts) and acoustic features of speech samples. Man-Whitney test was used for revealing differences between acoustic features of emotional speech samples correctly classified by experts. The aim of Regression analysis was to reveal the acoustic features of a child’s speech that could be considered as predictors for emotional state recognition by experts.

### Acoustic features of the stimulus material

Speech of Indian children is characterized by higher values of pitch than the speech of Russian children (Z-score = 9.119 p < 0.00001—Mann-Whitney test–for spontaneous speech, Z-score = 2.164 p < 0.03 –for acting speech). Spontaneous and acting speech of Russian children and spontaneous speech of Indian children in different emotional states is characterized by the pitch values of speech samples. The acting speech of Indian children is characterized by the intensity of pitch (Table 2).

**Table 2:** Acoustic features of spontaneous speech and acting speech in the stimulus material predict emotional state (Regression analysis data).

Type of speech	Language	Acoustic features	p	R^2^	β
**Spontaneous**	Russian	F0 F(1,88) = 6.152	0.01	0.069	0.026
		F0max F(1,88) = 5.749	0.01	0.061	0.247
		F0[max-min] F(1,88) = 7.503	0.007	0.061	0.248
	Tamil	F0 F(1,88) = 34.123	0.0000	0.279	0.528
		F0max F(1,88) = 27.421	0.0000	0.238	0.487
		F0min F(1,88) = 4.608	0.03	0.498	0.223
		F0[max-min] F(1,88) = 13.234	0.0004	0.130	0.362
**Acting**	Russian	F0 F(1,65) = 17.091	0.0001	0.208	0.456
		F0max F(1,65) = 9.52	0.002	0.128	0.357
		F0[max-min] F(1,65) = 6.97	0.01	0.083	0.311
	Tamil	Emax/Emin F(1,78) = 6.057	0.01	0.072	0.268
		Emax/E0 F(1,78) = 8.344	0.005	0.096	0.311

R^2^—correlation coefficient (R) squared; β—regression coefficient; p—is a number describing how likely it is that data would have occurred under the null hypothesis of statistical test

Types of emotional acting speech of Russian children are correlated with different acoustic features of speech (Table 3).

**Table 3:** The correlation between acoustic features of speech and types of emotional acting speech of Russian children (Multiple Regression analysis data).

R^2^	F	Independent variable	β	SE β	B	SE B	t (63)	p
**Dependent variable: Type of speech samples**								
	F (3,63) 15.693	Emax/E0	0.417	0.099	0.057	0.014	4.212	0.00008
		F0[max-min]	0.583	0.130	0.009	0.002	4.477	0.00003
		F0	-0.324	0.129	-0.006	0.002	-2.510	0.01

R^2^—correlation coefficient (R) squared; SE—standard error; β—standardized, B—unstandardized regression coefficients; p—is a number describing how likely it is that data would have occurred under the null hypothesis of statistical test

The native language (Russian—Tamil) is correlated with pitch values of spontaneous speech samples F(1,178) = 100.33 p < 0.00001 (R^2^ = 0.360 β = 0.059), and for acting speech–with intensity values: Emax/Emin F(1,77) = 4.576 p < 0.03 (R^2^ = 0.031 β = 0.175), with E0min/E0 F(1,145) = 4.928 p < 0.03 (R^2^ = 0.033 β = -0.181). The gender of Russian children does not affect the acoustic features of speech samples. The gender of Indian children is correlated with the minimum values of pitch (the boys had higher pitch values than the girls (p < 0.001 –Mann-Whiney test).

## Results

### Perceptual data

#### Study 1. Spontaneous speech

*Russian speaking children’s spontaneous speech*. Russian experts recognized the state of joy (60% of correct answers) and neutral (84%) in the speech of Russian speaking children better vs the state of sadness (44%) and anger state (25%). They attributed the largest number of speech samples to a neutral state (Table 4). Indian experts recognized the emotional state of anger (46%) better than Russian experts; they classified the state of joy (39%), the state of sadness (36%), and neutral state (46%) in the speech of children worse than Russian experts did. Russian experts recognized the emotional state of Russian children better vs Indian experts (p < 0.0001 –Mann-Whitney test)–particularly for the neutral state (p < 0.0001) and joy (p < 0.01), but not for sadness and anger.

**Table 4:** Confusion matrix for emotion classification in the spontaneous speech of Russian children by Russian and Indian experts.

	Russian experts					Indian experts			
	Joy	Neutral	Sad	Anger		Joy	Neutral	Sad	Anger
**Joy**	**60**	36	3	1	**Joy**	**39**	25	18	18
**Neutral**	9	**84**	6	1	**Neutral**	7	**46**	31	16
**Sadness**	5	45	**44**	6	**Sadness**	13	31	**36**	20
**Anger**	9	47	19	**25**	**Anger**	11	22	21	**46**
**Total**	83	212	72	33	**Total**	70	124	106	100
**Recall**	0.6	0.84	0.44	0.25	**Recall**	0.39	0.46	0.36	0.46
**Precision**	0.72	0.40	0.61	0.76	**Precision**	0.56	0.37	0.34	0.46
**F1-score**	0.66	0.54	0.51	0.38	**F1-score**	0.46	0.41	0.35	0.46
Unweighted Average Recall (UAR) - 0.53					Unweighted Average Recall (UAR) - 0.42				

The number of experts’ answers (%) correctly assigned to the corresponding category of emotions is highlighted

Agreement between Russian experts in recognizing all emotional states of Russian children was moderate (k = 0.418), between Indian experts was fair (k = 0.335), between both groups was fair (k = 0.218). Russian and Indian experts agreed on the joy state (k = 0.407, moderate strength of agreement) via a Russian child’s spontaneous speech. Agreement of experts in each language group in determining the state of joy (k = 0.52—for Russian experts, k = 0.508—for Indian experts) was higher than recognizing other emotional states.

*Tamil speaking children’s spontaneous speech*. Indian experts recognized the neutral state (86% of correct answers), sadness (86%), anger (81%), and joy (80%) in the speech of Tamil children (Table 5).

**Table 5:** Confusion matrix for emotion recognition in the spontaneous speech of Tamil speaking children by Russian and Indian experts.

	Russian experts					Indian experts			
	Joy	Neutral	Sad	Anger		Joy	Neutral	Sad	Anger
**Joy**	**42**	35	8	15	**Joy**	**80**	17	2	1
**Neutral**	11	**72**	10	7	**Neutral**	6	**86**	6	2
**Sadness**	7	31	**58**	4	**Sadness**	1	9	**86**	4
**Anger**	24	29	5	**42**	**Anger**	5	11	3	**81**
**Total**	84	167	81	68	**Total**	92	123	97	88
**Recall**	0.42	0.72	0.58	0.42	**Recall**	0.80	0.86	0.86	0.81
**Precision**	0.50	0.43	0.72	0.62	**Precision**	0.87	0.70	0.89	0.92
**F1-score**	0.46	0.54	0.64	0.5	**F1-score**	0.88	0.77	0.87	0.86
Unweighted Average Recall (UAR) - 0.54					Unweighted Average Recall (UAR) - 0.83				

Russian experts recognized the neutral state (72%) and the state of sadness (58%) in the speech of Tamil speaking children better vs the states of joy and anger (42%). When recognizing all emotions in the spontaneous speech of Tamil children, the agreement between Indian experts (k = 0.644) was higher than between Russian experts (k = 0.352), between groups of Russian and Indian experts the strength of agreement was fair (k = 0.331). Russian and Indian experts agreed on the sadness state (k = 0.512, moderate strength of agreement) via a Tamil child’s spontaneous speech. Agreement between Indian experts was found for recognizing the state of sadness (k = 0.725), anger (k = 0.723), joy (k = 0.66), between Russian experts in recognizing the state of sadness (k = 0.522).

The average recognition recall (UAR) of the emotional state from the spontaneous speech of Russian children for Russian experts was 0.53; for Indian experts– 0.42; UAR for spontaneous Tamil speech for Russian experts—0.54, for Indian experts– 0.83. Experts poorly recognized emotional states of children from spontaneous speech, while were more accurate in recognizing the emotional states of children in their native language. Agreement between experts between groups was fair.

#### Study 2. Acting speech

*2*.*1*. *Words reflecting the emotional states*. ***Russian speech*.** Russian experts classified all emotional states of Russian children with high accuracy (85%) (Table 6). Indian experts better recognized the state of anger (97% of correct answers), the state of sadness (85% of correct answers), and worse–the neutral state (30%). The average recognition accuracy of the emotional state for Russian experts was 88.9 ± 6%; for Indian experts was 66.7 ± 32.8%.

**Table 6:** Confusion matrix for emotion recognition in the emotional words of Russian children by Russian and Indian experts.

	Russian experts					Indian experts			
	Joy	Neutral	Sad	Anger		Joy	Neutral	Sad	Anger
**Joy**	**85**	5	10	0	**Joy**	**40**	45	5	10
**Neutral**	5	**90**	5	0	**Neutral**	0	**30**	70	0
**Sadness**	0	5	**95**	0	**Sadness**	5	10	**85**	0
**Anger**	0	3	10	**87**	**Anger**	0	3	0	**97**
**Total**	90	103	120	87	**Total**	45	88	160	107
**Recall**	0.85	0.90	0.95	0.87	**Recall**	0.4	0.3	0.85	0.97
**Precision**	0.94	0.87	0.79	1.00	**Precision**	0.89	0.34	0.53	0.91
**F1-score**	0.89	0.89	0.86	0.93	**F1-score**	0.55	0.32	0.65	0.94
Unweighted Average Recall (UAR) - 0.89					Unweighted Average Recall (UAR) - 0.63				

Agreement between Russian experts in recognizing all emotions was substantial (k = 0.714), for Indian experts was moderate (k = 0.556), between Russian and Indian experts was moderate (k = 0.462). Russian and Indian experts agreed on the state of anger (k = 0.812, almost perfect), and their opinions were closer to the sadness state (k = 0.415) than to the state of joy (k = 0.35) and the neutral state (k = 0.113). The highest agreement between Russian experts was determined for the state of anger (k = 0.752) and joy (k = 0.726), between the Indian experts—for the state of anger (k = 0.855).

***Tamil speech***. Russian experts better classified the state of anger (100% of correct answers), worse–a joy state (55%) (Table 7). Indian experts better recognized the neutral state (90% of correct answers), and worse–the joy state (80%). The average recognition accuracy of the emotional state for Russian experts was 77.5 ± 18.3%, for Indian experts was 85 ± 10.7%.

**Table 7:** Confusion matrix for emotion recognition in the emotional words of Tamil children by Russian and Indian experts.

	Russian experts					Indian experts			
	Joy	Neutral	Sad	Anger		Joy	Neutral	Sad	Anger
**Joy**	**55**	25	10	10	**Joy**	**80**	10	10	0
**Neutral**	10	**75**	15	0	**Neutral**	0	**90**	0	10
**Sadness**	0	20	**80**	0	**Sadness**	5	10	**85**	0
**Anger**	0	0	0	**100**	**Anger**	5	5	5	**85**
**Total**	65	120	105	110	**Total**	90	115	100	95
**Recall**	0.55	0.75	0.80	1.00	**Recall**	0.8	0.9	0.85	0.85
**Precision**	0.85	0.63	0.76	0.91	**Precision**	0.89	0.78	0.85	0.89
**F1-score**	0.67	0.68	0.78	0.95	**F1-score**	0.84	0.84	0.85	0.87
Unweighted Average Recall (UAR) - 0.78					Unweighted Average Recall (UAR) - 0.85				

Agreement among Russian experts in recognizing all emotions of Tamil children by the words was moderate (k = 0.519), for Indian experts was substantial (k = 0.64), between Russian and Indian experts was moderate (k = 0.575). The agreement between the Russian experts (k = 0.898) and between the Indian experts (k = 0.677) was the highest in recognizing the state of anger vs recognizing other emotions, experts of the two groups agreed on the state of anger (k = 0.775), sadness (k = 0.588), and neutral state (k = 0.487).

UAR of the emotional state from words reflecting the emotional states of Russian children for Russian experts was 0.89; for Indian experts– 0.63; UAR for Tamil speech for Russian experts—0.78, for Indian experts– 0.85.

*2*.*2*. *Words and phrases reflecting the emotional states*. ***Russian speech*.** Russian experts better classified the state of joy and anger (90% of correct answers), worse–a neutral state (80%) via Russian children’s emotional words and phrases (Table 8). Indian experts better recognized the state of anger (90% of correct answers), and worse–the neutral state (40%). The average recognition accuracy of the emotional state for Russian experts was 87.1 ± 10.5%; for Indian experts was 64.1 ± 26.0%. Russian experts recognized the emotional state of Russian children in the emotional words and phrases of Russian children better vs. Indian experts (p < 0.01 –Mann-Whitney test)–particularly for the neutral state (p < 0.05) and joy (p < 0.05).

**Table 8:** Confusion matrix for emotion recognition in the emotional words & phrases of Russian children by Russian and Indian experts.

	Russian experts					Indian experts			
	Joy	Neutral	Sad	Anger		Joy	Neutral	Sad	Anger
**Joy**	**90**	5	5	0	**Joy**	**50**	38	2	10
**Neutral**	3	**80**	17	0	**Neutral**	2	**40**	58	0
**Sadness**	0	13	**87**	0	**Sadness**	2	28	**70**	0
**Anger**	0	2	8	**90**	**Anger**	4	6	0	**90**
**Total**	93	100	117	90	**Total**	58	112	130	100
**Recall**	0.90	0.80	0.87	0.90	**Recall**	0.50	0.40	0.70	0.90
**Precision**	0.97	0.80	0.74	1.00	**Precision**	0.86	0.36	0.54	0.90
**F1-score**	0.93	0.80	0.80	0.95	**F1-score**	0.63	0.38	0.61	0.90
Unweighted Average Recall (UAR) - 0.87					Unweighted Average Recall (UAR) - 0.63				

Consistency between Russian experts in recognizing all emotions of Russian children according to words and phrases was substantial (k = 0.705), with a greater agreement in recognizing emotions of different valences and high activation—anger (k = 0.827) and joy (k = 0.825). For the Indian experts, moderate agreement (k = 0.47) for all emotions, and the highest (substantial) for the state of anger (k = 0.761) was found. Moderate agreement was determined between Russian and Indian experts (k = 0.469) for all emotions. Russian and Indian experts agreed on the state of anger (k = 0.789) and joy state (k = 0.475) more than on the state of sadness (k = 0.396) and neutral state (k = 0.148).

***Tamil speech.*** Russian experts better classified the state of anger (100% of correct answers), worse–a neutral state (62.5%) (Table 9). Indian experts better recognized the state of anger (90% of correct answers), and worse–the neutral state (85%). The average recognition accuracy of the emotional state for Russian experts was 75.6 ± 21.6%, for Indian experts was 87.5 ± 10.0%; Indian experts recognized the emotional state of Tamil children in the emotional words and phrases of better vs. Russian experts (p < 0.05 –Mann-Whitney test)–for neutral state and joy.

**Table 9:** Confusion matrix for emotion recognition in the emotional words & phrases of Tamil children by Russian and Indian experts.

	Russian experts					Indian experts			
	Joy	Neutral	Sad	Anger		Joy	Neutral	Sad	Anger
**Joy**	**65**	15	5	15	**Joy**	**87.5**	7.5	5	0
**Neutral**	17.5	**62.5**	20	0	**Neutral**	7.5	**85**	2.5	5
**Sadness**	0	25	**75**	0	**Sadness**	5	7.5	**87.5**	0
**Anger**	0	0	0	**100**	**Anger**	2.5	5	2.5	**90**
**Total**	82.5	102.5	100	115	**Total**	102.5	105	97.5	95
**Recall**	0.65	0.63	0.75	1.00	**Recall**	0.88	0.85	0.875	0.9
**Precision**	0.79	0.61	0.75	0.87	**Precision**	0.85	0.81	0.90	0.95
**F1-score**	0.71	0.62	0.75	0.93	**F1-score**	0.86	0.83	0.89	0.92
Unweighted Average Recall (UAR) - 0.76					Unweighted Average Recall (UAR) - 0.88				

Agreement between Russian experts in recognizing all emotions of Tamil children by words and phrases was moderate (k = 0.553), for Indian experts was substantial (k = 0.695), between Russian and Indian experts was moderate (k = 0.59). The highest agreement between Russian experts was found for the state of anger (k = 0.868), between Indian experts—when determining the emotions of anger (k = 0.799) and sadness (k = 0.733)—emotions of the same valence and different activation. Russian and Indian experts agreed on the state of anger (k = 0.808), sadness (k = 0.6), joy state (k = 0.522), and neutral state (k = 0.406).

UAR of the emotional state from words and phrases reflecting the emotional states of Russian children for Russian experts was 0.87; for Indian experts– 0.63; UAR for Tamil speech for Russian experts—0.76, for Indian experts– 0.88. Experts’ recognition of emotions from emotional words, words and phrases was high. Agreement between expert groups was moderate. Both groups of experts well recognized the state of anger with a significant agreement.

*2*.*3*. *Meaningless text*. ***Russian speech*.** Russian experts better recognized a state of joy and sadness (98% and 85% of correct answers), worse—a neutral state (65%) via Russian children’s acting speech. Indian experts better recognized the state of sadness, anger, and joy (88%, 85%, and 70%), worse recognized the neutral state (48%) (Tables 10 and 11). Both groups of experts determined the state of sadness equally well (85% and 88% of the answers of Russian and Indian experts; recall—0.85 & 0.88). The average recognition accuracy of the emotional state for Russian experts was 79.4 ± 18.4%; for Indian experts was 72.5 ± 31.3%.

**Table 10:** Confusion matrix for emotion classification in the meaningless text of Russian children by Russian and Indian experts.

	Russian experts					Indian experts			
	Joy	Neutral	Sad	Anger		Joy	Neutral	Sad	Anger
**Joy**	**98**	2	0	0	**Joy**	**70**	25	0	5
**Neutral**	5	**65**	30	0	**Neutral**	10	**48**	42	0
**Sadness**	0	15	**85**	0	**Sadness**	0	12	**88**	0
**Anger**	17	10	3	**70**	**Anger**	10	5	0	**85**
**Total**	120	92	118	70	**Total**	90	90	130	90
**Recall**	0.98	0.65	0.85	0.7	**Recall**	0.70	0.48	0.88	0.85
**Precision**	0.82	0.71	0.72	1.0	**Precision**	0.78	0.53	0.68	0.94
**F1-score**	0.89	0.68	0.78	0.82	**F1-score**	0.74	0.51	0.77	0.89
Unweighted Average Recall (UAR) - 0.80					Unweighted Average Recall (UAR) - 0.73				

**Table 11:** Confusion matrix for emotion classification in the meaningless text of Tamil children by Russian and Indian experts.

	Russian experts					Indian experts			
	Joy	Neutral	Sad	Anger		Joy	Neutral	Sad	Anger
**Joy**	**67**	17	11	5	**Joy**	**56**	29	14	1
**Neutral**	19	**70**	11	0	**Neutral**	40	**54**	4	2
**Sadness**	14	23	**62**	1	**Sadness**	14	36	**48**	2
**Anger**	10	6	0	**84**	**Anger**	7.5	7.5	0	**85**
**Total**	110	116	84	90	**Total**	117.5	126.5	66	90
**Recall**	0.67	0.70	0.62	0.84	**Recall**	0.56	0.54	0.48	0.85
**Precision**	0.61	0.60	0.74	0.93	**Precision**	0.48	0.43	0.73	0.94
**F1-score**	0.64	0.65	0.67	0.88	**F1-score**	0.51	0.48	0.58	0.89
Unweighted Average Recall (UAR) - 0.71					Unweighted Average Recall (UAR) - 0.61				

Moderate agreement between Russian experts in recognizing all the emotions of Russian children in the meaningless text (k = 0.592), a substantial agreement between Indian experts (k = 0.631), and moderate—between Russian and Indian experts (k = 0.536) were revealed. The greatest agreement was shown between Russian experts in determining joy (k = 0.759), within the group of Indian experts for the state of anger (k = 0.728). Russian and Indian experts agreed on the state of anger (k = 0.623), sadness (k = 0.652) and joy state (k = 0.562), the strength of agreement was less for neutral state (k = 0.261, fair).

***Tamil speech.*** Russian experts better recognized the state of anger (84% of correct answers), worse—the state of sadness (62%) via Tamil children’s acting speech. Indian experts better recognized the state of anger (85%), worse recognized the state of sadness (48%) (Table 11). Both groups of experts determined the state of anger equally well (84% and 85% of the answers of Russian and Indian experts; recall—0.84 & 0.85). The average recognition accuracy of the emotional state for Russian experts was 70.3 ± 26.9%; for Indian experts was 60.6 ± 31.7%. There were no significant differences between Russian and Indian experts in emotion classification in the meaningless text.

Moderate agreement between Russian experts in recognizing all emotions of Tamil children from meaningless texts (k = 0.544), for Indian experts (k = 0.503), between Russian and Indian experts (k = 0.471) was revealed. Both groups of experts agreed in responses to anger (k = 0.748) with the greatest consistency and within the expert groups (k = 0.716 for Russian experts, k = 0.754 for Indian experts), sadness (k = 0.522) and joy (k = 0.437), the strength of agreement was less for the neutral state (k = 0.21).

UAR of the emotional state from the meaningless text of Russian children for Russian experts was 0.80; for Indian experts– 0.73; UAR for Tamil speech for Russian experts—0.71, for Indian experts– 0.61. Experts’ recognition of emotions by meaningless text was worse than by emotional words, words and phrases. Consistency between expert groups was moderate.

The data on Cohen kappa statistic for all perceptual experiment are presented in the Table 12.

**Table 12:** Expert’s agreement in recognizing the emotional states of children via speech: Within a language group and between groups (Cohen kappa statistic).

Type of speech	Language	Emotions	Russian experts	Indian experts	Russian & Indian experts
Spontaneous: words & phrases	Russian	joy	0.52	0.508	0.407
		neutral	0.404	0.216	0.186
		sadness	0.255	0.323	0.078
		anger	0.162	0.269	0.074
		all emotions	0.481	0.335	0.218
	Tamil	joy	0.206	0.66	0.245
		neutral	0.317	0.527	0.512
		sadness	0.522	0.725	0.512
		anger	0.407	0.723	0.337
		all	0.352	0.644	0.331
Acting: Emotional words	Russian	joy	0.726	*	0.35
		neutral	0.697	0.171	0.113
		sadness	0.673	0.629	0.415
		anger	0.752	0.855	0.812
		all	0.714	0.556	0.462
	Tamil	joy	*	0.548	0.409
		neutral	0.346	0.642	0.487
		sadness	0.490	0.661	0.588
		anger	0.898	0.677	0.775
		all	0.519	0.64	0.575
Acting: Emotional words & phrases	Russian	joy	0.825	0.325	0.475
		neutral	0.564	0.135	0.148
		sadness	0.56	0.499	0.396
		anger	0.827	0.761	0.789
		all	0.705	0.47	0.469
	Tamil	joy	0.497	0.662	0.522
		neutral	0.300	0.588	0.406
		sadness	0.509	0.733	0.6
		anger	0.868	0.799	0.808
		all	0.553	0.695	0.59
Acting: Meaningless texts	Russian	joy	0.759	0.688	0.562
		neutral	0.387	0.376	0.261
		sadness	0.589	0.696	0.652
		anger	0.578	0.728	0.623
		all	0.592	0.613	0.536
	Tamil	joy	0.544	0.49	0.437
		neutral	0.356	0.29	0.21
		sadness	0.57	0.525	0.522
		anger	0.716	0.754	0.748
		all	0.544	0.503	0.471

Note

*- the formula for Cohen kappa statistic does not allow calculating the average values due to zeros in answers of one of the experts

### Acoustic features of the speech of Russian and Indian children correctly recognized by Indian and Russian experts

Recognition of the emotional state of children corresponding to the expert’s nationality and language is associated with the following acoustic features of emotional speech (Table 13. When recognizing the emotional state of children by their spontaneous speech, Russian and Indian experts rely on the pitch values of speech samples: average, maximum, minimum. Indian experts use fewer acoustic features of acting speech for classification the emotional state of children vs Russian experts.

**Table 13:** Acoustic features of child speech predictors for the recognition of the emotional states (Regression analysis).

**Speech**	**Emotional state via speech**	**Acoustic features**	**p**	**R^2^**	**β**
**Spontaneous**	Russian children	Russian experts			
		F0 F(1,43) = 9.986	0.002	0.188	0.434
		F0max F(1,43) = 4.707	0.035	0.099	0.314
		F0[max-min] F(1,43) = 4.473	0.040	0.094	0.307
		Indian experts			
		F0 F(1,13) = 6.141	0.027	0.328	0.566
		F0max F(1,13) = 6.893	0.020	0.346	0.589
		F0[max-min] F(1,60) = 10.487	0.001	0.148	0.386
	Indian children	Russian experts			
		F0 F(1,27) = 17.833	0.000	0.398	0.630
		F0max F(1,27) = 12.977	0.001	0.325	0.570
		F0[max-min] F(1,27) = 6.755	0.01	0.200	0.447
		Indian experts			
		F0 F(1,60) = 30.317	0.0000	0.336	0.579
		F0max F(1,60) = 31.068	0.0000	0.341	0.584
		F0min F(1,60) = 6.585	0.01	0.099	0.314
**Acting**	Russian children	Russian experts			
		F0 F(1,57) = 12.384	0.001	0.179	0.422
		F0max F(1,57) = 8.017	0.006	0.123	0.351
		F0[max-min] F(1,57) = 6.080	0.017	0.096	0.310
		E0min/E0 F(1,57) = 8.385	0.005	0.128	-0.358
		Emax/Emin F(1,57) = 10.418	0.002	0.155	0.393
		Indian experts			
		E0min/E0 F(1,38) = 6.2359	0.017	0.141	-0.376
	Indian children	Russian experts			
		F0min F(1,42) = 7.3986	0.010	0.150	-0.387
		F0[max-min] F(1,42) = 12.370	0.001	0.227	0.477
		Emax/E0 F(1,42) = 9.0533	0.004	0.177	0.421
		Emax/Emin F(1,42) = 6.9171	0.012	0.141	0.376
		Indian experts			
		F0min F(1,55) = 7.9255	0.007	0.126	-0.355
		F0[max-min] F(1,55) = 11.948	0.001	0.179	0.423
		Emax/E0 F(1,55) = 4.0579	0.050	0.069	0.262

Significant differences between acoustic features of the emotional speech of Russian children correctly classified by Russian experts and acoustic features of the emotional speech of Indian children correctly classified by Indian experts were revealed. Russian experts identified (range 0.75–1.0) a state of joy and a neutral state when listening to the spontaneous speech of Russian children. The pitch values and range of pitch values lower in speech samples indicated neutral state vs joy state. Indian experts correctly classified (range 0.75–1.0) neutral state, joy, sadness, and anger states when listening to the spontaneous speech of Indian children. Speech samples reflected the emotional states of Indian children varied on the values of pitch, pitch maximum, and minimum values, and pitch range. The states of joy and anger don’t differ significantly based on analysis of acoustic features of speech (Table 14). The acoustic features of acting emotional speech of Russian children don’t differ significantly for states of sadness and anger. The states anger and joy vary in the range of pitch values. The acoustic features of acting emotional speech of Indian children don’t differ significantly for states of sadness and neutral, joy and anger.

**Table 14:** Significant differences between acoustic features of the emotional speech of Russian children correctly (range 0.75–1.0) classified by Russian experts and acoustic features of the emotional speech of Indian children correctly classified by Indian experts.

Types of speech	Acoustic features	Russian	p	Indian	p
**Spontaneous**	F0	N < J	0.0015	N < J	0.002
				N < A	0.0009
				S < J	0.007
				S < A	0.0006
	F0max			N < J	0.0004
				N < A	0.0006
				S < J	0.0001
				S < A	0.0001
	F0min			N < S	0.01
				N < J	0.03
				N < A	0.01
	F0[max-min]	N < J	0.03	N < J	0.009
				S < J	0.0005
				S < A	0.0007
**Acting**	F0	N < S	0.017	S < J	0.005
		N < J	0.0004	S < A	0.015
		N < A	0.00002		
	F0max	N < J	0.0009	N < J	0.029
		N < A	0.002	S < J	0.001
		S < J	0.017	S < A	0.009
	F0min			A < N	0.025
	F0[max-min]	N < J	0.001	N < J	0.02
		N < A	0.007	N < A	0.011
		S < J	0.014	S < J	0.008
		A < J	0.047	S < A	0.005
	Emax/E0			N < J	0.039
				N < A	0.024
	E0min/E0	N < A	0.021		
	Emax/Emin	N < J	0.018		
		N < A	0.009		

R–Speech samples of Russian children correctly classified by Russian experts; I—Speech samples of Indian children correctly classified by Indian experts; p–is a number describing how likely it is that data would have occurred under the null hypothesis of Mann-Whitney test; N–neutral state, J—joy, S—sadness, A—anger

Based on these acoustic features of the speech, the auditory system of the Indian and Russian experts made it possible to classify speech samples as reflecting the corresponding emotional state “joy–neutral–sadness–anger”.

Anger state is characterized by the shorter duration of speech samples (vs joy state p = 0.003—Mann-Whitney test, sadness state p = 0.003, neutral state p = 0.016). The pitch values and energy distributions are not differing significantly from the joy state.

Joy state is characterized by the highest values of F0max (vs anger state p = 0.0009, sadness state p = 0.0002, neutral state p = 0.016), and pitch range F0[max-min] (vs anger state p = 0,001; sadness state p = 0.0000, neutral state–p = 0.003), and longer duration of speech samples (vs anger state p = 0.003, sadness state p = 0.003, neutral state p = 0.016). The correct recognition of joy state of children by Russian and Indian experts is correlated with the type of speech material F(1,26) = 9.2542 p < 0.005 (R^2^ = 0.2625 β = -0.5123).

Sadness state is characterized by the lower values of pitch (vs anger state p = 0.04, joy p = 0.01); lower values of F0max (p < 0.05—Mann-Whitney test); least range values (p < 0.05). The values of E0max/E0min are less vs anger and joy state at the trend level. Accurate recognition of sadness state of children by Russian and Indian experts is correlated with the language (Russian-Tamil) of children F(1,35) = 7.1974 p < 0.01 (R^2^ = 0.1706 β = 0.413), speech material F(1,35) = 11.132 p < 0.002 (R^2^ = 0.2413 β = 0.4912), child’s gender–for boys better vs girls F(1,35) = 4.914 p<0.03 (R^2^ = 0.1231 β = 0.3509).

The neutral state is characterized by the lower values of pitch energy E0min/E0 and values of E0max/E0min are less vs anger, joy at the trend level; and pitch values vs anger and joy states at the trend level.

## Discussion

The results of the study showed the ability of cross-cultural recognition of the emotional state via speech of children belonging to different language environments. The native Russian and Tamil speaking experts were more accurate in recognizing the emotional states of children in their native language, especially on speech, which allows experts to rely on the linguistic characteristics, along with acoustic ones. This fact has been noted by other researchers who point out that, although basic emotion recognition is universal, emotion recognition is more accurate when speakers and receivers come from the same culture than in other cultures [4, 5, 21].

Our data on Russian and Tamil languages is confirmed by the results of the cross-cultural study on English and Hindi listeners [12], vocal emotions recognition in Spanish, Chinese, Arabic, and English speech by English listeners [15], cross-cultural study of the emotional tone of voice recognition by Chinese and British native speakers [41]. We carried out a comparative analysis of the recognition of the four emotional states “joy—neutral-sadness -anger” in Russian and Tamil children via speech by experts from two languages—Russian and Tamil. The selected emotions are following the Neurocultural theory of emotion [3]. Basic emotions have similar neurobiological mechanisms [42], but their implementation is determined by the culture and society from the standpoint of the paradigm of social constructivism [43]. This conception does not deny the contribution of biological systems to emotional syndromes, but the author supposes that the functional significance of emotional responses is to be found largely within the sociocultural system. The presence of mechanisms of occurrence for each of the types of basic emotions such as joy, sadness, anger, regardless of the usual environment and cultural environment, also determines the presence of features based on which a particular emotional state can be recognized.

Four types of speech material were used–spontaneous speech, emotional words, words and phrases and meaningless sentences. The native Russian and Tamil speaking experts were more accurate in recognizing the emotional states of children in the acting speech vs. spontaneous speech. Using spontaneous speech, Russian experts recognize the emotional state of Russian children worse, compared to Indian experts who determine emotions from the speech of Tamil children. Russian experts recognize the neutral state and joy state and Indian experts classified neutral state and anger by a spontaneous speech of Russian children better than other emotional states. Both groups of experts had better consent classifying the neutral and joy state for Russian children with the moderate agreement between the experts. This fact may be related to the cultural peculiarities of the emotions expressed by Russian children when interacting with an adult. 8–12 years old children do not manifest negative emotions in a dialogue with an adult, try to respond neutrally, or demonstrate positive emotions, that was shown for Russian children aged 6–7 years [44]. For Western culture, it was shown that between 6 and 10 years of age, elementary school children show an increasing awareness of emotions’ display rules [45, 46].

Indian experts recognize all the emotional states via spontaneous speech of Tamil children, while Russian experts determined neutral state and sadness. Russian and Indian experts agreed on the sadness via a Tamil child’s speech. The manifestation of emotions in the spontaneous speech of Indian children is more expressive in comparison with Russian peers that are correlated with higher values of voice pitch—the basic feature of emotional speech [20]. Therefore, it can be assumed that sadness, as an emotion of weak activation, was recognized by Russian experts better than emotions of high activation and different valence (joy and anger), recognizing which Russian experts gave the same number of correct answers.

When selecting options for emotional acting speech, we assumed that emotional words and phrases that linguistically correspond to different emotional states will allow native speakers to recognize emotions based on voice and linguistic features, and non-native speakers only on the basis of voice. As a universal speech material that allows experts to rely only on voice features, we used the meaningless sentence, following other researchers [20, 27].

Both groups of experts recognize the state of anger via acting speech with a high agreement. Why did experts from different cultures identify the emotion of anger so well? This emotion is vital. Anger is considered a very arousing emotional condition. Ch. Darwin in his book “The Expression of Emotions in Man and Animals” (1872) described the greatest similarity in the expression of the emotion of anger in animals and humans [7]. Anger is critical to motivating action and approaching and is considered a survival response inherent in all living things [28]. People experiencing anger daily and consider it one of the most typical examples of emotion [47]. Traditional Tamil culture gives is special characterized anger expression in terms of its manifestation, suppression, and management. History literature gives suitable examples of considering anger as a prime component of valor. In military environments, anger expression and aggression are a way to compensate for feelings such as inferiority, shame, hurt, irritation, and unassertiveness [48, 49]. Research shows that anger is expressed by children and adults in order to gain acceptance in society [50]. In the United States (US), there’s an emphasis on the value of positive emotions—such as happiness or pride, Russian culture values all emotions—including negative emotions were shown in a comparative study of parents in Russia and the US and children’s literature. Russian parents are more likely than US parents to read stories to their children that contain negative emotions such as fear, anger, and sadness [51]. In our study speech samples correctly classified by Indian and Russian experts as characterized anger state of children is characterized by emphasized acoustic pattern—the shorter duration of speech samples and high values of pitch (but not differing significantly from the joy state).

Along with a high recognition by both groups of experts of the state of anger through acting speech, Russian experts better recognized joy state in the speech of children of the same nationality, and Indian experts–sadness, joy, and neutral states in the speech of Tamil children. Our findings are supported by the results of another study that examined how individual emotions (anger, disgust, fear, sadness, happiness, pleasant surprise, and neutrality) are recognized and acoustically differentiated in four linguistic contexts—English, German, Hindi, and Arabic. While overall recognition scores varied across languages, anger, sadness, and fear were generally best recognized regardless of language [52].

An interesting finding was the better recognition by Russian experts of the emotional state of Tamil children from meaningless texts. Apparently, Russian experts, who have extensive experience in recognizing emotions in the speech of Russian children who weakly manifest their state during interaction with adults, transferred their knowledge to the speech of Tamil children. This is consistent with the position of the dialect theory, according to which individuals tend to judge other people’s responses based on their cultural style [23]. From the point of view of Indian experts, the meaningless texts spoken by Tamil children were less emotional than their spontaneous speech, which made it difficult to determine emotions. This assumption is supported by the data that Indian experts use fewer acoustic features of actor’s speech (minimum pith values, pitch range, maximum intensity ratio) compared to Russian experts to classify the emotional state of Tamil children according to speech.

The similar acoustic features of the emotional speech of Russian and Indian children correctly assigned by Russian and Indian experts to the corresponding emotions were identified. The acoustic features of the emotional speech of children correspond to those in the speech of adults [53, 54]. Higher pitch values are usually associated with high-arousal emotions angry and happiness, while lower pitch values are more associated with low-arousal emotions such as sadness [36]. Happy and angry states are related with a very wide range of pitch values compared to neutral speech while sadness state is associated with a less wide range of pitch values [54]. When analyzing the acting speech, the values of the pitch intensity influenced the correct recognition of the state of sadness. It was noted that higher energy is usually associated with high-arousal emotions such as anger and happiness, while lower energy is more associated with low-arousal emotions such as sadness [35], which corresponds to the data obtained in our study. Speech signals with explicit differences in acoustic patterns were more accurately classified by experts as belonging to emotions of different activation. The pitch values and intensity of acting speech and the pitch values of spontaneous speech are important for recognition the emotional states of children by experts. For the spontaneous speech of Indian children, the pitch values change more significantly depending on the emotional state vs spontaneous speech of Russian children. Our results confirm the data on the importance of the main frequency of the speaker for the transmission of vocal emotions in different languages [53].

However, we cannot make a global conclusion concerning all Russian and Indian children. We considered the Tamil language and the region of residence of Tamil children and experts—Vellore and Russian language in the specific region of Russia–St. Petersburg, which is closer to Europe than to Asia in its cultural and historical traditions.

The study raised questions regarding the relationship between the type of emotional speech and its recognition, and the experience of experts. Further research will shed light on these questions. In general, our data on the recognition of emotions in children’s speech based on the material of two distant languages showed that, despite the universality of basic emotions, on the one hand, the cultural environment affects their expression and perception, on the other hand, there are universal non-linguistic acoustic features of the voice that allow us to identify emotions via speech.

## Conclusion

It is shown that Russian and Indian experts are capable to recognize correctly the emotional states of Indian and Russian children by their speech, but with varying accuracy. Experts poorly recognized emotional states of children from spontaneous speech, while were more accurate in recognizing the emotional states of children in their native language. Agreement between experts between groups was fair. According to the spontaneous speech, Russian and Indian experts better determine the neutral state for Russian children, but a moderate agreement between the experts of both groups for the state of joy was determined, and neutral and sad states from the speech of Tamil children, with moderate agreement on sadness. The native Russian and Tamil speaking experts were more accurate in recognizing the emotional states of children in the acting speech vs. spontaneous speech. Both groups of experts recognize the state of anger via acting speech with a high agreement. The difference between the groups of experts was in the definition of joy, sadness, and neutral states depending on the test material with a different agreement. The agreement between the experts of the two groups was the highest when recognizing the emotional state of children by words and phrases and by words in comparison with meaningless texts. Native speakers of Russian and Tamil languages more accurately recognized the emotional states of children in their native language via the all speech material, with the exception of meaningless text, when Russian experts classified the emotional state of Tamil children better than Indian experts. The unweighted average recall is higher for acting speech than for spontaneous speech. The Indian and Russian experts relied on similar acoustic features while determining corresponding emotions via speech of Russian and Tamil children. It was shown that for recognition of emotional states via children’s acting speech by experts, the pitch values and intensity are important; the pitch values are important for emotions recognition via spontaneous speech. For the spontaneous speech of Indian children, the acoustic features (the pitch–average, maximum, minimum, and range) change more significantly depending on the emotional state vs spontaneous speech of Russian children. Differences in the acoustic characteristics of the speech of children referred by two groups of experts to different emotional states are described.

## Supporting information

S1 Data (PDF)

S2 Data (PDF)

S3 Data (XLSX)

S4 Data (XLSX)

S5 Data (XLSX)

S6 Data (XLSX)

S7 Data (PDF)

S8 Data (XLSX)

S9 Data (XLSX)

S10 Data (XLSX)
