# Supplementary material for: The children’s emotional speech recognition by adults: Cross-cultural study on Russian and Tamil language
Source: PLoS One. 2023 Feb 15;18(2):e0272837. doi: 10.1371/journal.pone.0272837 (PMC9931107; doi:10.1371/journal.pone.0272837)
Supplement: S1 Data — (PDF) [file pone.0272837.s001.pdf]

Instructions to the participants

The original instructions were in Russian for Russian experts, in Tamil for Indian experts, we provide English translations here.

The aim of the auditory perceptual experiment is to examine the ability of Russian and Indian experts to recognize the state of Russian and Indian children by their speech. The judgments are recorded by instructing participants to select the most appropriate label from a fixed list of options: neutral state, sadness, joy, anger.

Your participation is anonymous: we are not collecting or storing names, email addresses, or other data that would allow us to identify the participants. Other data (age, gender, results) will be permanently stored by the researchers and can later be used for analysis and publication. You will be assigned a personal number, which we will use when processing the questionnaires.

You will be asked to listen to tests containing samples of children's speech. Before listening, please fill in the fields in the questionnaire, mark your gender, age, professional work experience, the presence of your own children (their age) and the experience of interacting with children (children of friends, other relatives in the family).

Then, please put on your headphones. Turn on test playback. Listen one time and mark in the questionnaire opposite the number of the corresponding signal, what emotional state of the child corresponds to the listened signal.

You can get information about the results of the test from the project coordinator (information is provided in the Informed consent to participate in the study) by sending him your identification number.
