# Supplementary material for: The children’s emotional speech recognition by adults: Cross-cultural study on Russian and Tamil language
Source: PLoS One. 2023 Feb 15;18(2):e0272837. doi: 10.1371/journal.pone.0272837 (PMC9931107; doi:10.1371/journal.pone.0272837)
Supplement: S2 Data — (PDF) [file pone.0272837.s002.pdf]

Stimulus material

<u>Words for emotions:</u>

JOY: Joy, Beautiful, Cool, Super
NEUTRAL: Nothing, Normal, Well, and Three
SADNESS: Sad, Sadness, Longing, Hard, Sad, Cry
ANGER: Scream, Bite, Break, Crush, Beat, Push, Stupidly, Scold

<u>Phrases for emotions:</u>

JOY: I love when everything is beautiful!
NEUTRAL: I'm fine
SADNESS: It's raining outside, I'm sad. Sad time
ANGER: I love to beat and break everything. Crush and bite when everyone got me. I am annoyed by everything.

<u>Meaningless text</u>
Russian children
1. The fragment of the poem "Jabberwocky" by L. Carroll (1872)

Twas brillig, and the slithy toves
Did gyre and gimble in the wabe:
All mimsy were the borogoves,
And the mome raths outgrabe.
On Russian:
Варкалось. Хливкие шорьки
Пырялись по наве,
И хрюкотали зелюки,
Как мюмзики в мове.

2. In Russian, there is an artificial phrase based on the Russian language, in which all morphemes are replaced by meaningless combinations of sounds

“Гло́кая ку́здра ште́ко будлану́ла бо́кра и курдя́чит бокрёнка”
(L.V. Shcherba).

Tamil children
1. **In Tamil: (Heyman et al., 2007)**

தாடி நீண்ட தாத்தா தடவி ஒருநாள் பார்த்தார்: மாடப் புறா ரண்டு மைனாப் பறவை ரண்டு காடைக் குருவி ரண்டு கரிக் குருவியும் ரண்டு தாடிக்குள்ளே தங்கி கூடு கட்டி அங்கு மூடி ஒளிந்து கொண்டு முட்டை யிட்டன ரண்டு!

It reads like:

Thaadi Neenda thaathaa thadavi orunaal paarthaar: maada pura randu, mynaa Paravai randu, kaadai kuruvi randu, kari kuruviyum randu, thaadikullay Thaangi koodu katti angu moodi olinthu kondu motai ittana randu!

Meaning in English:

When grandpa stuck his finger Deep into his beard He found many strange things there
The strangest things you've heard:

Out came a turtle dove, Not just one, but two; In flew a sunbird Not just one, but two
A yellow bird has got inside, And a blackbird too.
They'll make their cozy nests in there, And lay their eggs inside them
And dear old Grandpa's long white beard, Will quite completely hide them.

2. “அக்கர தக்கத்து டக்கரா சக்கரு வமதும்மே லிப்பாசிலா....”

This sentence reads like:

“akkarae thakkathu dakkarrae chakkaru vamathummae libbashela….”
