# Supplementary material for: The children’s emotional speech recognition by adults: Cross-cultural study on Russian and Tamil language
Source: PLoS One. 2023 Feb 15;18(2):e0272837. doi: 10.1371/journal.pone.0272837 (PMC9931107; doi:10.1371/journal.pone.0272837)
Supplement: S7 Data — (PDF) [file pone.0272837.s007.pdf]

Table. Expert's agreement in recognizing the emotional states of children via speech: within a language group and between groups (Cohen kappa statistic)

Type of speech	Language	Emotions	Russian experts	Indian experts	Russian & Indian experts
Spontaneous: words & phrases	Russian	joy	0.52	0.508	0.407
		neutral	0.404	0.216	0.186
		sadness	0.255	0.323	0.078
		anger	0.162	0.269	0.074
		all emotions	0.481	0.335	0.218
	Tamil	joy	0.206	0.66	0.245
		neutral	0.317	0.527	0.512
		sadness	0.522	0.725	0.512
		anger	0.407	0.723	0.337
		all	0.352	0.644	0.331
Acting: Emotional words	Russian	joy	0.726	*	0.35
		neutral	0.697	0.171	0.415
		sadness	0.673	0.629	0.415
		anger	0.752	0.855	0.812
		all	0.714	0.556	0462
	Tamil	joy	*	0.548	0.409
		neutral	0.346	0.642	0.487
		sadness	0.490	0.661	0.588
		anger	0.898	0.677	0.775
		all	0.519	0.64	0.575
Acting: Emotional words & phrases	Russian	joy	0.825	0.325	0.475
		neutral	0.564	0.135	0.148
		sadness	0.56	0.499	0.396
		anger	0.827	0.761	0.789
		all	0.705	0.47	0.469
	Tamil	joy	0.497	0.662	0.522
		neutral	0.300	0.588	0.408
		sadness	0.509	0.733	0.600
		anger	0.868	0.799	0.808
		all	0.553	0.696	0.59
Acting: Meaningless texts	Russian	joy	0.759	0.688	0.561
		neutral	0.387	0.376	0.261
		sadness	0.589	0.696	0.652
		anger	0.578	0.728	0.623
		all	0.592	0.613	0.536
	Tamil	joy	0.544	0.49	0.437
		neutral	0.356	0.29	0.21
		sadness	0.57	0.525	0.522
		anger	0.716	0.754	0.748
		all	0.544	0.503	0.471

*- the formula for Cohen kappa statistic does not allow calculating the average values due to zeros in answers of one of the experts
